# Ion Channels in Odor Information Processing of Neural Circuits of the Vertebrate Olfactory Bulb

**DOI:** 10.3390/ijms252413259

**Published:** 2024-12-10

**Authors:** Yunqing Yu, Ping Liao, Ruotian Jiang

**Affiliations:** 1 Department of Anesthesiology, West China Hospital, Sichuan University, Chengdu 610041, China; 2 Laboratory of Anesthesia and Critical Care Medicine, National-Local Joint Engineering Research Center of Translational Medicine of Anesthesiology, West China Hospital, Sichuan University, Chengdu 610041, China

**Keywords:** olfactory disorders, ion channels, olfactory bulb, odor signal transduction

## Abstract

Olfactory disorders and their associated complications present a considerable challenge to an individual’s quality of life and emotional wellbeing. The current range of treatments, including surgical procedures, pharmacological interventions, and behavioral training, frequently proves ineffective in restoring olfactory function. The olfactory bulb (OB) is essential for odor processing and plays a pivotal role in the development of these disorders. Despite the acknowledged significance of ion channels in sensory functions and related pathologies, their specific involvement in OB remains unexplored. This review presents an overview of the functions of various ion channel families in regulating neuronal excitability, synaptic transmission, and the complex processes of olfactory perception. The objective of this review was to elucidate the role of ion channels in olfactory function, providing new insights into the diagnosis and treatment of olfactory dysfunction.

## 1. Introduction

Correct odor perception is of great consequence for a multitude of aspects of life, including food intake, safety, hygiene, and social interaction. It has been demonstrated to positively impact the quality of life, cognitive function, and mental wellbeing. Nevertheless, olfactory disorders affect approximately 20% of the population, frequently due to aging, post-viral infections, nasal and sinus conditions, neurodegenerative diseases, and head injuries [1,2,3,4]. The objective of treatment is to address the underlying cause, which may entail the use of nasal corticosteroids, antibiotics, and surgical intervention for conditions such as sinusitis or head trauma [1,3]. Olfactory training, which involves daily exposure to different odors for several months, has been demonstrated to yield only partial recovery rates, with numerous patients failing to regain their sense of smell [2,3,5].

The olfactory bulb (OB) serves as the primary processing center for the olfactory system. The olfactory bulb plays a pivotal role in odor encoding, encompassing the identification, intensity, and temporal aspects of odors. Furthermore, it is integral to odor learning, memory, and processing [6,7,8,9,10,11]. Besides its central role in normal olfactory function, the OB has also been implicated in various olfactory disorders. Disruption of OB function may result from several mechanisms, including neuronal infection, inflammation, and viral effects on axonal integrity. For example, in the context of the ongoing coronavirus pandemic, the OB may contribute to the development of parosmia, a condition characterized by distorted odor perception, through disrupted axon wiring [12,13]. Congenital anosmia, defined as the complete loss of sense of smell, can result from hypoplastic or aplastic OBs. Qualitative olfactory disorders may also involve the OB, potentially due to neuronal degeneration or regeneration [2,3,14]. A comprehensive understanding of the mechanisms underlying OB-related olfactory disorders is essential for developing effective diagnostic and therapeutic approaches.

Ion channels are important for the processing of sensory information and transmission of signals in several sensory modalities, including touch, pain, vision, hearing, and taste [15,16,17]. These channels regulate cellular excitability, synaptic transmission, and neuronal development, making them crucial targets for drug development. Although systematic reviews on ion channel functions exist for other sensory systems, such as the auditory, visual, and gustatory systems, the specific role of ion channels in olfactory information processing remains largely unexplored. In the auditory system, transient receptor potential (TRP) channels, large-conductance K^+^ channels (BK), and voltage-gated K^+^ channels (Kv) 7.1 and 7.4 play crucial roles in maintaining K^+^ cycling and sound transduction [18,19]. In the retina, Na^+^, Ca^2+^, cyclic nucleotide-gated (CNG), and K^+^ channels shape photoreceptor responses by converting photocurrents into voltage changes [20,21,22,23]. Similarly, taste perception depends on specific ion channels in taste receptor cells, including TRPM5 and otopetrin1 [24].

Mutations in ion channels have been shown to cause the loss or abnormalities of sensory function. For example, loss-of-function mutations in the SCN9A gene, which encodes Nav1.7, result in congenital insensitivity to pain and anosmia, while preserving innocuous sensation [25]. The most common association between mutations in a subunit of voltage-gated Ca^2+^ channels 1.4 (Cav1.4) and clinical presentation is incomplete congenital stationary night blindness and retinal cone dystrophy [21]. Moreover, mutations in KCNQ4, which encodes Kv7.4, are frequently associated with autosomal dominant nonsyndromic hearing loss [18]. A comprehensive understanding of the role of ion channels in olfactory information processing is essential for elucidating the underlying mechanisms of olfactory disorders and developing targeted therapeutic interventions.

This review emphasizes the underappreciated role of ion channels in the olfactory system, with a particular emphasis on the OB, which plays a pivotal role in olfactory signal modulation. The objective of this review was to present a comprehensive summary of recent advances in the distribution and function of ion channels in various neuronal types within the OB. The objective of this review is to enhance our understanding of the molecular mechanisms underlying olfactory information processing. The roles of these mechanisms in olfactory conduction will be explored, and potential therapeutic targets for olfactory disorders will be identified. This study provides novel insights into the diagnosis and treatment of olfactory dysfunction and associated diseases.

## 2. Neuron Types and Functions in the Olfactory Bulb

**Figure 1:**
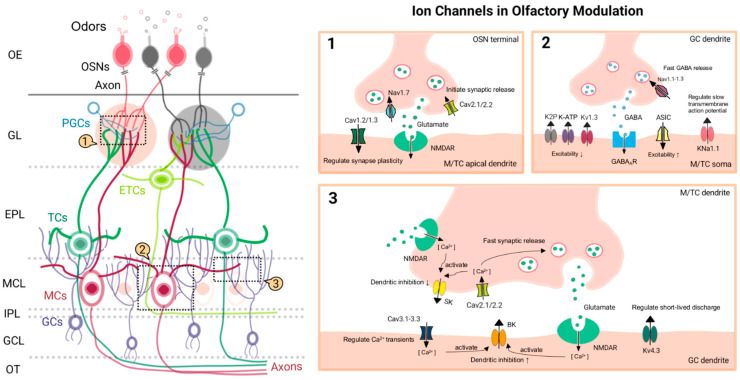
Left: Schematic representation of cellular architecture in the OB, from OSNs to M/TC axons, showing the interconnected cell types and neurotransmitter systems. Right: Primary synaptic transmission and ion channel function in the OB: 1. OSN terminal axons and M/TC apical dendrites; 2. GC dendrites and M/TC soma; 3. M/TC dendrite and GC dendrite. Abbreviations in the figure are listed in the table.

The complex distribution and specialized functions of neurons within the OB are essential for their precise role in modulating and transmitting olfactory information [8,9,11]. The OB is subdivided into six principal layers: olfactory sensory neuron (OSN) axon layer, glomerular layer (GL), external plexiform layer (EPL), mitral cell layer (MCL), internal plexiform layer (IPL), and granule cell layer (GCL). The principal cell types in the OB are glutamatergic neurons, including OSNs and mitral/tufted cells (M/TCs), which are responsible for transmitting olfactory information to higher-level processing centers. Furthermore, γ-aminobutyric acid (GABA) neurons, including granule cells (GCs) and select periglomerular cells (PGCs), regulate the balance between excitation and inhibition of mitral/tufted cells (M/TCs), contributing to odor processing and discrimination. In the nasal mucosa, OSNs detect chemical odor molecules via their receptors and transmit these signals via their axons to specialized synapses within the GL, which are organized according to distinct odor categories. The apical dendrites of M/TCs capture these signals, which subsequently propagate through their axons to the olfactory cortex. PGCs play a pivotal role in modulating the excitability of mitral/tufted cell (M/TC) clusters, maintaining an optimal signal-to-noise ratio, and facilitating accurate odor discrimination (Figure 1) [9,26,27]. Although short interneurons are widely distributed throughout the OB, their specific contributions remain poorly understood and are not the primary focus of this study.

### 2.1. OSNs

OSNs are essential bipolar cells that initiate chemical processing of odors and play a pivotal role in the olfactory system. The cilia of OSNs are within the nasal mucosa and contain receptors that facilitate the conversion of diverse chemical odor molecules present within the nasal cavity into electrical signals [28]. These electrical signals are conveyed through the cell body and axons of OSNs, ultimately reaching the OB. In the OB, axons extend into the glomeruli, forming clusters of synaptic connections with the dendrites of the M/TCs, facilitating efficient signal transmission [11]. These two fundamental structural characteristics facilitate odor discrimination. First, OSNs express distinct sets of odor receptors that respond to specific odors. Second, axons from OSNs of the same class form connections with glomeruli dedicated to particular odor categories [29]. This organization facilitates the refinement of odor perception intensity and enhances discrimination capabilities. Consequently, the synapses formed by OSNs in the OB represent the initial stages of olfactory transmission and are pivotal for accurate odor recognition.

### 2.2. Mitral/Tufted Cells

The transmission of olfactory information within the OB depends on the involvement of various neuronal types and synaptic connections. The primary excitatory projection neurons in the OB are MCs and TCs [9]. These neurons possess an apical dendrite that terminates in a dense tuft within the glomerular neuropil, as well as multiple lateral dendrites extending into the EPL. The cell bodies of MCs are in the deeper MCL, and their axons extend to specific downstream targets [30]. MC neurons are primarily excited through a multistep signaling pathway and play a crucial role in odor information processing by routing distinct odor signals to various regions of the olfactory cortex. Moreover, MCs are implicated in the encoding of odor identity and intensity in the olfactory cortex through complementary coding mechanisms [6,7]. They are responsible for integrating sensory inputs with behavioral states and adapting to changing environments, influencing olfactory sensitivity and behavior. In addition, TCs are involved in the processing of afferent olfactory sensory information in parallel with MCs, which encode complementary aspects of olfactory information. TCs exhibit greater sensitivity, broader tuning, lower response thresholds, concentration-invariant responses, and earlier responsiveness in the sniff cycle, rendering them more efficient in distinguishing similar odorants at low concentrations [26,30]. To discern the types of odors more accurately, external TCs establish an internal olfactory information connectivity system by extending axons from one glomerulus to GCs below another glomerulus. This direct connection links the glomerulus innervated by OSNs expressing the same olfactory receptors, effectively linking the source and neurons processing olfactory information [6,11].

The representation of odors in the OB depends on several mechanisms. In awake animals, the firing patterns of M/TCs convey more information about odor identity than their firing rates do. Phasic M/TCs demonstrate firing patterns synchronized with discrete phases of sniffing or respiration cycles [7]. This firing pattern is primarily influenced by local interneurons that provide regulatory feedback, shaping the excitability of M/TCs before the transmission of olfactory information to the piriform cortex [9,27]. These interneurons are indispensable for integrating sensory inputs with behavioral states and adapting to changing environments, which affect olfactory sensitivity and behaviors related to odor processing.

### 2.3. GCs

Inhibitory interneurons are indispensable elements of OB circuitry. The most prevalent inhibitory neurons in the OB are GCs, which lack axons but release the neurotransmitter GABA from their spiny apical dendrites. These dendrites interact with the lateral dendrites of excitatory M/TCs, regulating their activity in response to olfactory stimuli [9,27,31]. GCs display distinctive characteristics regarding synaptic organization. In contrast to the conventional unidirectional synaptic configuration, GCs form reciprocal synapses with the dendrites of other neurons, enabling both cell types to serve as presynaptic and postsynaptic elements [9,32]. The release of Glu from M/TC dendrites can stimulate postsynaptic GC dendrites, which subsequently releases GABA to exert inhibitory control over presynaptic M/TC dendrites, effectively reversing their synaptic roles.

GCs have traditionally been assumed to be involved in the recurrent and lateral inhibition of M/TCs, contributing to the coding of odor identity, modulating the output of M/TCs, and influencing olfactory behavior and detection [10]. Dendrodendritic synapses between M/TC and GC dendrites are of critical importance in this process. The release of glutamate from M/TC dendrites excites GC dendrites, which then inhibits M/TC dendrites via the release of GABA. This spatially localized self-inhibition of M/TCs and lateral inhibition contributes to odor discrimination [9,11,27,32].

### 2.4. Periglomerular Cells

Another significant category of inhibitory interneurons in the OB is PGCs. These cells have small cell bodies and limited dendritic branches and release the neurotransmitter GABA within the GL [9,11]. PGCs receive direct input from OSNs and use GABAB and D2 receptors to facilitate the presynaptic inhibition of sensory neurons. In addition, they receive indirect excitatory input from other neurons and provide inhibitory output to various cells in the GL, contributing to recurrent inhibition of activated M/TCs and odor-evoked suppression of M/TC firing [33]. PGCs exhibit a selective inhibitory effect on GCs, underscoring the intricate regulatory mechanisms governing olfactory information transmission [9].

Besides the intrinsic circuitry of the OB, the functions of local interneurons are modulated by a range of extrinsic factors, including metabolic peptides, neuropeptides, and hormones. Such extrinsic modulators may also influence olfactory processing and behavior. In addition, certain modulators, including dopamine, norepinephrine, and acetylcholine, have been observed to influence the function of ion channels, which play pivotal roles in developing and regulating the olfactory system [3,34,35].

## 3. Expression and Function of Ion Channels in the Olfactory Bulb

### 3.1. K^+^ Channels

#### 3.1.1. Voltage-Gated K^+^ Channels

The vertebrate Kv family, which encompasses 12 members spanning from Kv1 to Kv12, is classified based on amino acid sequence similarity and is distinguished by the presence of six transmembrane domains [36]. The expression of the Kv channel subunits varies among different cell types within the olfactory system. The Kv1.x subfamily, which represents the largest class of the Kv family, forms heteropolymers within the central nervous system. Specifically, six Kv1.x subunits (Kv1.1, Kv1.2, Kv1.3, Kv1.4, and Kv1.6) were identified as being expressed in the OB [37]. The distribution of these subunits varied across different neuronal substructures within the OB (Table 1).

Among the Kv1.x subunits, Kv1.3 has been extensively studied in the context of OB (Table 2). In a seminal study, Fadool et al. identified Kv1.3 as a significant contributor to the discharge of MCs in the OB [51]. Mice lacking Kv1.3 exhibited increased firing frequency in MCs, along with reduced levels of fasting blood glucose, insulin, and leptin. This physiological alteration results in enhanced olfactory sensitivity and discrimination as well as improved resistance to obesity [51,59]. Subsequent research has demonstrated that the deletion of Kv1.3 in early postnatal mice results in an increase in MC numbers [60], synaptic thinning in OSNs, and disrupted glomerular development. These changes are associated with increased anxiety and attention deficits owing to increased olfactory sensitivity [61,62]. The involvement of Kv1.3 in obesity is linked to its interactions with several key proteins, including insulin-dependent glucose transporter 4, insulin receptor kinases, and postsynaptic density protein-95. These interactions influence mitochondrial development and Kv1.3 phosphorylation in response to insulin [63,64].

It has been established that insulin and glucagon-like peptide-1, with Kv1.3 channel inhibitors, enhance the discharge of MCs [65,66]. Moreover, the active form of glucose has been demonstrated to directly activate Kv1.3, affecting the excitability of MCs [67]. Leptin has been demonstrated to suppress MC activity by activating K^+^ channels that bear a resemblance to Kv1.3. Nevertheless, further investigations are necessary to substantiate this relationship [34], and the specific cell-type functions of Kv1.3 in the OB remain incompletely understood. Most current studies have focused on MCs, which has restricted our understanding of the function of Kv1.3, which is also expressed in PGCs and GCs. Further research using cell-specific interventions must gain a more comprehensive understanding of the role of Kv1.3 in the OB function.

Besides the extensively studied Kv1.3 channel, other Kv channel subunits are widely expressed across different neuronal populations within the OB. Boda et al. observed elevated levels of Kv3.1 and Kv3.2 during the aging process, with Kv3.1 predominantly expressed in glomerular pericytes and implicated in rapid intraglomerular inhibition [68]. The Kv3.1 channel may facilitate a reduction in the firing frequency of projection neurons and an increase in peak time variability [33]. Kv3.4 is strongly expressed in the olfactory glomerulus, but it primarily colocalizes with OSN axons, and its precise role remains to be elucidated [37]. Kv1.3 and Kv3.1 serve as delayed rectifier channels, which are localized in the soma of MCs and PGCs, where they regulate cellular excitability [33,51]. In contrast, Kv3.4 functions as a rapidly inactivating channel in the presynaptic compartment of OSNs, where its rapid inactivation properties may modulate action potential intervals and influence the frequency of synaptic transmission [36,37].

The Kv4.2 subunit has been identified in the soma, lateral dendrites, apical dendrites, and intercellular junctions of M/TCs, whereas Kv4.3 has been detected in PGCs, deep short-axon cells, and GCs [42]. It has been demonstrated that brief transient responses observed in GCs depend on both AMPA receptor-mediated synaptic transmission and Kv4.3 inactivation [55]. In addition, other Kv channel subunits, including Kv10.1 and Kv10.2, are also extensively distributed within the OB [43,52]. Nevertheless, further investigation is required to fully understand their specific functions and contributions to olfactory information processing.

#### 3.1.2. Inward Rectifier K^+^ Channels

The inward rectifier K^+^ channel (Kir) family plays a pivotal role in the olfactory system and comprises four subunits, each featuring two transmembrane segments surrounding a pore-forming domain. In mammals, this family comprises 15 genes organized into seven subfamilies ranging from Kir1 to Kir7 [69].

In 1996, Horio et al. identified the prominent expression of Kir2.3 mRNA (also known as Irk3) across various regions of the olfactory system, including the OB, pre-olfactory nucleus, olfactory tubercle, and piriform cortex [70]. Subsequent studies have demonstrated the localization of specific Kir subunits within the OB. Kir2.1 is predominantly expressed in the GL, Kir2.3 is highly concentrated in the EPL, and Kir2.4 shows moderate levels in the MCL [44]. The activity of Kir currents in dopaminergic neurons surrounding the glomeruli is modulated by neurotransmitters that regulate neuronal excitability [71]. Sun et al. observed that oxytocin enhances adenosine triphosphate (ATP) production, which activates the K-ATP family (Kir6.1–6.2) in M/TCs. This increase in K-ATP activity results in a reduction in both spontaneous and odor-evoked neuronal firing, decreasing M/TC excitability. This improves the signal-to-noise ratio of odor responses and ultimately promotes the exploration of novel social stimuli [72,73]. In contrast to its effects on M/TCs, oxytocin has been observed to decrease odor-evoked responses in GCs of awake mice without altering their excitability. This suggests that oxytocin may distinctly affect M/TCs and GCs within the OB, which may be influenced by cell-specific expression of K-ATP.

#### 3.1.3. Two-Pore Domain K^+^ Channels

The two-pore domain K^+^ channel (K2P) family comprises 15 mammalian genes. These channels are distinguished by four transmembrane segments, exhibit slight rectification, and play a crucial role in establishing resting membrane potential, regulating cellular excitability, and enhancing K^+^ permeability in cells with specialized K^+^ transport requirements [74]. K2P channels have been demonstrated to play a pivotal role in a multitude of sensory functions. For example, in retinal ganglion cells, the K2P channel TASK-3 plays a significant role in maintaining intrinsic excitability by sensing synaptic proton release [23]. Similarly, in dorsal root ganglion (DRG) neurons, TASK3, TREK1, and TREK2 have been identified as regulators of neuronal excitability during pathological pain states [75,76,77].

Despite the widespread distribution of the K2P channel family in the OB, their specific role remains unclear. Six K2P family mRNAs, specifically Kcnk3, Kcnk9, Kcnk2, Kcnk10, Kcnk1, and Kcnk13, have been identified in OB [45]. The Kcnk3 and Kcnk9 genes exhibited high expression levels in the GL, whereas all six genes were present in olfactory GCs [45,78]. Adenosine A1 receptors (A1R) are important for the functioning of MCs. Upon binding to A1R, adenosine hyperpolarizes MCs by activating K2P, which suppresses spontaneous activity and enhances the signal-to-noise ratio, fine-tuning the input-output relationship of the sensory system [79]. However, previous studies have primarily used pharmacological methods to confirm that adenosine-evoked outward currents are mediated by K2P, leaving specific subtypes largely uncharacterized. Further research must distinguish and identify K2P subtypes that contribute to these currents. Additional research must elucidate the functional implications of K2P in the OB, particularly in the context of olfactory behavior and disease models. Schubert et al. demonstrated that A1R modulates dendrodendritic inhibition at the synaptic sites of MCs and GCs, affecting olfactory sensory information processing. Nevertheless, regulation of the A1R pathway does not appear to alleviate inflammation-induced hyperexcitability of MCs or restore olfactory function. This indicates that the potential protective effects of K2P and A1R signaling might not prevent hyperexcitability, Ca^2+^ overload, and neuronal injury during neuroinflammation [80].

#### 3.1.4. Ca^2+^-Activated and Na^+^-Activated K^+^ Channels

Ca^2+^-activated K^+^ channels (KCa) are activated by increases in intracellular Ca^2+^ levels. BK, also designated as KCa1.1, exhibited a high conductance of approximately 250 pS when activated in a symmetrical K^+^ environment at 100 mmol/L. In contrast, small-conductance K^+^ channels (SK) exhibit smaller conductance, ranging from 10 to 14 pS. These are further classified into three subtypes: KCa2.1, KCa2.2, and KCa2.3. SK and BK are involved in OB. SK is found in MCs but not GCs. Activation of these channels depends on Ca^2+^ influx through Cav and N-methyl-D-aspartate (NMDA) auto receptors in dendrites. The blockade of SK currents has been observed to enhance action potential discharge and induce dendritic inhibition [53]. In contrast, activation of the N-methyl-D-aspartate receptor (NMDAR) in GC dendrites mediates short-term inhibition through the influx of calcium ions coupled with the activity of the BK channel [56]. The distinct subcellular functions of these channels are closely related to their refined olfactory modulation capabilities, which illustrates the complex interplay of ion channels in the processing of olfactory information within the vertebrate OB.

Historically, Na^+^-activated K^+^ channels (KNa) have been classified within the BK channel family owing to their substantial conductance and structural similarity. However, recent studies have shown these channels are primarily regulated by fluctuations in cytoplasmic Na^+^ and chloride levels in mammals rather than by Ca^2+^ activation [81]. KNa was observed to be widely distributed throughout the OB, with Slick/KNa1.2, which exhibited the highest expression levels in the plexiform layer, and Slack/KNa1.1 being predominantly found in the GL. GCs express Slick channels in both somata and processes that extend into the MCL, facilitating the formation of dendrodendritic synapses [49]. Research has indicated that Slack, particularly in M/TCs, exhibits substantial delayed outward currents during normal olfactory transmission [50]. Moreover, slack plays a crucial role in compensating for outward currents in MCs lacking Kv1.3, contributing to slow transmembrane action potential saturation. This compensation may explain the abnormal olfactory phenotype observed in mice lacking Kv1.3 [82].

### 3.2. Voltage-Gated Ca^2+^ Channels

Cav families are classified according to their molecular structure and current characteristics [83]. The α1 subunit of the Ca^2+^ channel functions as the primary pore-forming component and acts as a voltage sensor. In contrast, the β subunit interacts with the cytoplasmic domain of the α1 subunit along with other auxiliary subunits to collectively regulate channel activity. Studies on neurons have identified several calcium channel subtypes due to different α1 subunits: Cav1.1-Cav1.4, the molecular counterpart of L-type Ca^2+^ channels (LTCCs); Cav2.1, the molecular counterpart of P/Q-type Ca^2+^ channels (P/QTCCs); Cav2.2, the molecular counterpart of N-type Ca^2+^ channels (NTCCs); Cav2.3, the molecular counterpart of R-type Ca^2+^ channels (RTCCs); and Cav3.1–3.3, the molecular counterpart of T-type Ca^2+^ channels (TTCCs). Among these, P/QTCCs, NTCCs, LTCCs, and RTCCs are classified as high-voltage-activated channels, whereas TTCCs are classified as low-voltage-activated channels. These channels are highly expressed in the OB, except for RTCCs.

#### 3.2.1. LTCCs

In the brain, approximately 90% of LTCCs (Cav1.1-Cav1.4) are Cav1.2, with the remaining 10% being Cav1.3. These two subtypes are typically co-expressed within the same neuron and contribute to modulating postsynaptic firing and Ca^2+^-dependent signaling pathways involved in regulating gene expression, a process known as excitation-transcription coupling [84]. Ca^2+^ currents mediated by Cav1.2 and Cav1.3 are distinguished by their long-lasting nature and exhibit a distinctive property of slow inactivation and require substantial depolarization for activation. LTCCs play a role in several processes, including learning, memory formation, drug addiction, and neuronal development [85].

In the OB, LTCCs have been identified in the apical dendrites of neonatal rat MCs, where they are involved in synaptic release and interactions with NMDAR. This interaction facilitates Ca^2+^-dependent plasticity during the initial stages of odor preference learning, which is mediated by protein kinase A [54]. Furthermore, the LTCC blockade has been demonstrated to inhibit the induction of long-term potentiation in primary OB slices, providing additional evidence for their involvement in this process [86]. In addition, LTCCs are involved in memory processing in MCs, following the receipt of inputs from the piriform cortex. LTCCs perform different functions on young adult and aged animals. In the initial stages of olfactory learning, norepinephrine, acting through β-adrenoceptors, stimulates cAMP production, which leads to LTCC phosphorylation, enhances Ca^2+^ influx, and promotes long-term memory formation [87]. However, in aged organisms, excessive Ca^2+^ influx through LTCCs has been linked to reactive oxygen species (ROS) generation and neuronal death, which may contribute to cognitive decline and memory impairment [88]. Based on these findings, LTCCs may represent a promising target to treat age-related cognitive and memory-related disorders.

#### 3.2.2. P/QTCCs

P/QTCCs (Cav2.1) have been shown to play a role in the release of neurotransmitters. P/QTCCs have been observed to exhibit robust labeling in the PGCs of the OB and in a subset of neurons distributed throughout the deep layers of the entorhinal and piriform cortices [46]. The Cav2.1 subunit is localized to the presynaptic terminals of OSN axons in the OB, where it defines a subset of the glomeruli. The localization of Cav2.1 affects synaptic release, action potential patterns, and the regulation of other Ca^2+^-dependent channels, influencing the processing of olfactory signals [38].

The initial transformation of odor signals occurred at the synaptic glomeruli. Ion channels, including Nav1.7, Cav2.1, and Cav2.2, are localized to the presynaptic terminals of OSNs and serve as “switches” for neurotransmitter release in response to olfactory stimuli. Similarly, the lateral dendrites of M/TCs exhibit a rapid neurotransmitter release facilitated by Cav2.1 and Cav2.2, which activates a network of GABAergic interneurons, initiating olfactory modulation. The shared characteristics of Cav2.1 and Cav2.2 at the presynaptic terminals of OSNs and M/TCs suggest a potential consistency in channel distribution and function among projection neurons in the OB. This is likely influenced by chemotactic factors and their differential expression during development [89].

#### 3.2.3. NTCCs

Similarly, NTCCs (Cav2.2) are located in the glomerular neuropils of both primary and accessory OBs, where they colocalize with OSN axon terminals [90]. NTCCs exhibit rapid inactivation and require robust depolarization for their activation. Primarily located in presynaptic membranes, they serve as the initial trigger for the release of neurotransmitters [85]. In mice lacking Cav2.2, the efficacy of excitatory postsynaptic currents (EPSCs) in M/T cells is enhanced, indicating that the Ca^2+^ channel subunits at these synapses undergo compensatory changes. The absence of Cav2.2 has been observed to result in a reduction in paired-pulse depression at the OSN-M/T cell synapses, with a concomitant decrease in peak current amplitudes in response to paired stimulation. This has been associated with alterations in social behaviors, including hyper-aggressiveness [39]. N- and P/Q-type Ca^2+^ channels in mitral cell dendrites, which are essential for rapid neurotransmitter release, are activated upon arrival of an action potential [32,91]. Calcium channels with disparate voltage-activation characteristics can fulfill disparate functional roles within the same cellular context. In the OB: High-voltage-activated Ca^2+^ channels situated at the dendritic spines of GCs are responsible for generating single action potentials following Ca^2+^ influx [91]. In contrast, low-voltage-activated TTCCs are primarily responsible for regulating subtle Ca^2+^ transients in the GC dendrites. These TTCC-mediated Ca^2+^ dynamics assist in maintaining cellular excitability in response to action potentials and facilitate lateral inhibition by modulating voltage-dependent Ca^2+^ signaling [57]. The diversity of Ca^2+^ channel characteristics and their distribution within a single-cell type enables the precise modulation of Ca^2+^ signaling and regulation of specific cellular processes.

#### 3.2.4. TTCCs

TTCCs (Cav3.1–3.3) exhibit rapid inactivation and are readily activated by minimal depolarization. They are primarily detected in cardiac and smooth muscle cells and are responsible for the electrical pacemaker activity. In situ hybridization of mouse brain sections demonstrated substantial levels of TTCCs and CaV3.1–3.3 transcripts in the OB [92,93], with prominent labeling in the olfactory nerve layer and glomeruli, and moderate levels in the MCL and GCL [47]. The functional properties of TTCCs, including their low activation threshold, fast voltage-dependent inactivation, rapid recovery, and slow deactivation [83], indicate that they may play a role in regulating Ca^2+^ influx and signaling within the OB, which is a critical component of olfactory sensory processing and information transmission [57]. TTCCs play a role in regulating Ca^2+^ transients in dendrites, particularly in response to action potentials, and contribute to lateral inhibition by modulating voltage-dependent Ca^2+^ dynamics in GCs [47,57].

#### 3.2.5. Ca^2+^ Modulators

Besides the fundamental role of Ca^2+^ channels in electrophysiological processes, several auxiliary Ca^2+^ modulators contribute significantly to OB function. The α2-δ-1 subunit has been identified in the human olfactory system M/TCs, suggesting its involvement in odorant perception and processing [90]. This subunit plays a crucial role in enhancing Ca^2+^ channel functionality by facilitating the trafficking of α1 subunits to the cell membrane, increasing the number of functional channels available [94].

### 3.3. Voltage-Gated Na^+^ Channels

Voltage-gated Na^+^ channels (Nav) are indispensable for initiating action potentials in electrically excitable cells. This family of proteins is encoded by genes designated Nav1.1–Nav1.9 [95]. In both mice and humans, four specific isoforms (Nav1.1, Nav1.2, Nav1.3, and Nav1.7) are encoded by genes located on chromosome 2 and exhibit substantial clustering in both species [96]. These Nav isoforms were functionally expressed in the OB. Adult-born GCs in the OB express voltage-gated sodium channels (Nav) 1.1–1.3, which are essential for preserving their characteristic morphological traits and neurotransmission. Urban et al. demonstrated that siRNA-mediated knockdown of Nav1.1–1.3 resulted in a reduction in dendritic spines and hindered dendritic complexity in GCs [58]. Na^+^ imaging may indicate a subcellular distribution of Nav channels that are enriched in the MC axons and GCs apical dendrites: action potential trains generated smaller Na^+^ signals in the MC lateral dendrites compared to those in axons; in the GC apical dendrites, action potential trains induced Na^+^ transients without detectable single spikes [97]. Moreover, the Nav1.2 isoform is essential for normal dendritic GABA release and rapid recognition of similar odors. GCs express Nav1.2 in dendritic spines, and its deletion has been demonstrated to impair spiking, GABA release, and mitral cell inhibition, resulting in delayed odor discrimination [40]. Mice with a heterozygous deletion of Nav1.1 display aversion to novel food and social odors. Treatment with a GABAA receptor-positive allosteric modulator has been shown to rescue this abnormal social behavior, indicating a connection with impaired GABAergic neurotransmission [98].

The localization of Nav to dendritic spines is also of great importance for providing precise temporal feedback inhibition to MCs. The activation of these channels within spines has been demonstrated to enhance Ca^2+^ influx and postsynaptic depolarization while leaving NMDAR signaling unaltered [99]. Moreover, the Nav1.7 isoform has been identified at the axon terminals of OSNs [41,100]. In contrast to Nav1.5, which is predominantly expressed in the dendritic knob of nasal mucosal epithelium, Nav1.7 exhibits a markedly smaller ‘window current’ and decreased spontaneous channel opening during the resting membrane state, suggesting distinct functional characteristics of Nav channels in the olfactory system [101]. Mice in which Nav1.7 is conditionally deleted in the OSNs cannot initiate synaptic signaling from their axon terminals and display deficiencies in odor perception and related behaviors. These findings underscore the importance of Nav1.7 in synaptic signaling within the olfactory system [102].

As the sole population of adult-born neurons in the OB, GCs express specific ion channels. The Na^+^ channel family, particularly Nav1.1–1.3, plays a vital role in synaptic differentiation and conduction of GCs, supporting their dendritic development and neural signaling. As renewable neurons in the mature central nervous system, the development and chemotactic functions of GCs progressively become dysregulated with age. Dysregulation can lead to hyposmia or anosmia [2,4,58]. Therefore, Nav1.1–1.3 may serve as an important target for studies on olfactory aging and related disorders.

### 3.4. Other Channels

#### 3.4.1. Cyclic Nucleotide-Gated Ion Channels

Cyclic adenosine monophosphate (cAMP) and cyclic guanosine monophosphate (cGMP) directly activate a class of non-selective channels known as CNG channels. Six genes encode proteins that form CNG channels. These comprise four alpha subunits (CNGA1–CNGA4) and two beta subunits (CNGB1 and CNGB3). These CNG ion channels typically form heterotetrameric complexes, often comprising two or three subunits, and serve as the primary pathways for Ca^2+^ influx into cells [103,104]. In the olfactory system, the activation of olfactory receptors by odorant molecules initiates a cascade of events, including activation of the cAMP signaling pathway and CNG channels, which ultimately results in the depolarization of OSNs in the olfactory epithelium [105]. In addition, research indicates that cGMP functions as a secondary messenger in the neural networks of the OB, particularly through the CNGA2 and CNGA3 subunits [48,103,106,107].

The CNGA3 subunit is predominantly expressed in the principal cells of the OB, including OSN axons, mitral and tufted cells, and interneurons such as PGCs, short-axon cells, glial cells, and immature migrating neurons [48]. Both CNGA2 and CNGA3 are present in the axons of OSNs and contribute to the processing of distinct odor information. A particular group of glomeruli, designated as “necklace glomeruli”, receives substantial cholinergic innervation and is innervated by OSNs that express the CNGA3 subunit. These glomeruli, which remain intact in CNGA2-deficient mice, play a crucial role in mediating responses to specific odorants [10]. Cyclic nucleotides can bidirectionally modulate synaptic transmitter release from OSN axons, exhibiting both facilitative and depressive effects at varying concentrations [108]. This differential modulation may underlie the potential mechanism by which CNGA2 and CNGA3 discriminate specific odorants. Moreover, the Ca^2+^/calmodulin (Ca^2+^/CaM) complex inhibits CNG channels in OSNs, affecting their cyclic nucleotide sensitivity and contributing to their sensory adaptation. The subunit composition of the CNG channel differs between olfactory and retinal rod cells, leading to disparate Ca^2+^/CaM-dependent regulatory mechanisms [103].

The CNGB1b subunit, in conjunction with the CNGA2 and CNGA4 subunits, binds cyclic nucleotides and plays a critical role in the activation of heterotetrameric olfactory CNG channels. Additionally, the CNGB1b subunit accelerates the deactivation of these channels, facilitating the rapid termination of odorant signals in OSNs [109].

#### 3.4.2. Acid-Sensing Ion Channels

Acid-sensing ion channels (ASICs) are ligand-gated channels activated by extracellular protons. To date, six ASIC isoforms have been identified. The ASIC family comprises six isoforms: ASIC1a, ASIC1b, ASIC2a, ASIC2b, ASIC3, and ASIC4. Among these, ASIC1a, heteromeric ASIC1a/2a, and ASIC3 were expressed in the M/TCs [110,111]. Furthermore, ASIC1a is present within the glomeruli of the OB [112]. Activation of these ASICs has been demonstrated to result in depolarization and increased intracellular Ca^2+^ levels in vitro. ASIC1-deficient mice exhibit prolonged latency in locating buried food, augmented sniffing time in response to acidic odors, and aberrant avoidance behavior toward specific odors relative to their wild-type counterparts [111]. These findings underscore the importance of ASICs in olfactory function. Nevertheless, it remains unclear whether these behavioral abnormalities are mediated by M/TCs, OSNs, and PGCs in the glomeruli, or a combination of these cell types.

In the central nervous system, ASICs modulate neuronal excitability in response to changes in extracellular pH [16]. Mice that received intranasal application of zinc gluconate, an inhibitor of ASIC1a, exhibited a pronounced increase in latency to uncover buried food. These findings indicate that the olfactory abnormalities observed in ASIC1-deficient mice may be because of odor receptors on the nasal mucosa rather than the OB itself. The expression of various ASIC isoforms in different cell types within the OB, coupled with their roles in modulating neuronal excitability and olfactory behavior, highlights the significance of ASICs in olfactory information processing. Further research must elucidate the precise mechanisms by which ASICs contribute to odor perception and discrimination within the olfactory neural circuits.

**Table 2:** Function and potential clinical relevance of ion channels in the olfactory bulb.

Channels	Function	Potential Clinical Relevance
K^+^ channels		
Kv1.3	Reduce firing frequency in MCs [51] Involved in the OB development [60]	Abnormal olfactory sensitivity and discrimination in Kv1.3-deficient mice [51,61,62]; glucose metabolism and obesity [51,59,63,64,65,66,67]; human polymorphism rs2821557 (T/C) [113]
Kv3.1	Reduce firing frequency and increase peak time variability of projection neurons [33]	
Kv4.3	Modulate brief transient responses in GCs [55]	
Kir2.1	Modulate neuronal excitability in PGCs [71]	
K-ATP	Decrease M/TC excitability and improve the signal-to-noise ratio of odor responses [72,73]	Novel social exploration [73]
K2P	Decrease spontaneous activity in MCs [79]	Adenosine-related olfactory processing [79,80]
SK	Enhance dendritic inhibition in MCs [53]	
BK	Mediate short-term inhibition in the GC dendrites [56]	
Slick	Facilitate the formation of the GC dendrites [49]	
Slack	Exhibits delayed outward currents in MCs [50]	Olfactory sensitivity and discrimination [82]
Ca^2+^ channels		
LTCCs	Facilitate Ca^2+^-dependent plasticity of the MC apical dendrites [54]	Odor preference learning [54];Long-term memory formation [86,87]
P/QTCCs	Modulate synaptic release in the OSN axons [38]	
NTCCs	Modulate synaptic release in the OSN axons and GC dendrites [32,91]	Social behaviors including hyper-aggressiveness [39]
TTCCs	Regulate subtle Ca^2+^ transients in the GC dendrites [57]	
Na^+^ channels		
Nav1.1–1.3	Preserve morphological traits and neurotransmission in adult-born GCs [58]	Odor discrimination [40] and response to novel food and social odors [98]
Nav1.7	Initiate synaptic signaling in the OSN axons [102]	Alteration of olfactory sensitivity in human rs41268673C>A and rs6746030C>T alleles [114,115] and mutation (c.3734A>G, p.N1245S) [116]
Other channels		
CNGA2		Isolated congenital anosmia in human stop mutation (c.634C>T, p.R212*) [117] and (c.577C>T, p.A193*) [118]
CNGA3	Mediate specific odorant processing in the OSN axons [10]	
CNGB1	Facilitate the rapid termination of odorant signals in OSNs [109]	Diminished or absent olfactory function in seven human CNGB1 mutations [119]
ASICs		Abnormal olfactory sensitivity and discrimination in ASIC1-deficient mice [111]

## 4. Genetic Mutations and Dysfunctions of Ion Channels in Odor-Related Disorders

Odor-related disorders can be classified into two broad categories: quantitative and qualitative disorders [120]. Quantitative disorders are defined as alterations in the capacity to detect and identify odors. The subdivision of these disorders may be based on the results of the smell identification tests, which include hypersensitivity, reduction (hyposmia), and complete loss of smell (anosmia). While most olfactory disorders are acquired, sometimes individuals are born without a sense of smell, a condition medically defined as congenital anosmia. In these patients, the OB is typically hypoplastic or aplastic, and the olfactory sulcus is shallow [121]. In contrast, qualitative olfactory dysfunction is characterized by distorted or phantom-odor perceptions. Parosmia is defined as a distorted odor perception in the presence of an odor source, whereas phantosmia is defined as an odor perception in the absence of an odor. Qualitative olfactory disorders are frequently precipitated by sinus or upper respiratory tract infections, as well as head trauma [122]. Qualitative olfactory disorders manifest during periods of neuronal degeneration or regeneration [4]. Phantom odors have been linked to psychiatric and neurological disorders [14].

The treatment approach for odor-related disorders typically focuses on addressing underlying causes. In cases of sinusitis or head trauma, the recommended treatment approach typically involves the use of nasal corticosteroids, antibiotics, or surgical interventions [1,3]. Although various therapeutic agents, including theophylline, vitamin A, and α-lipoic acid, have been evaluated in clinical trials, olfactory training has been demonstrated to be the only effective therapeutic intervention. This approach entails deliberate olfactory stimulation through inhalation of various odors on multiple occasions throughout the day, a practice that has been demonstrated to have a beneficial impact [5].

Further studies of the genetic mutations and ion channel dysfunction associated with odor-related disorders may yield valuable insights that could lead to clinical improvements. Mutations in Kv1.3, Nav1.7, CNGA2, and CNGB1 have been identified as potential causes of a specific type of olfactory disorder. Consequently, therapies aimed at restoring the normal functions of these ion channels have been explored. This may entail pharmacological intervention, gene therapy, or other strategies to address the underlying causes of dysfunction.

### 4.1. Kv1.3

The olfactory system and its variability play a significant role in the regulation of body weight [123,124,125]. This influence is not limited to the quality and quantity of food intake; it also affects energy and basal metabolism, partly through the activity of the K^+^ channel, Kv1.3. This clinical evidence supports the findings of the animal studies. The rs2821557 mutation in the Kv1.3 gene has been associated with alterations in glucose homeostasis and olfactory sensitivity. Individuals with this mutation exhibit a hyperolfactory phenotype and a lower body mass index than the general population [113]. The functionally relevant polymorphism rs2821557 (T/C) in the Kv1.3 gene has been linked to alterations in glycemic homeostasis and olfactory sensitivity [113,126,127]. The major allele, T, is associated with a “super-smeller” phenotype, lower plasma glucose levels, and resistance to diet-induced obesity compared to the minor allele, C. This phenotype is analogous to that observed in Kv1.3-deficient mice [51]. It is noteworthy that the results also demonstrated a sex-dependent effect, with heterozygous females exhibiting superior performance compared with heterozygous males [126]. These findings highlight the intricate relationships among individual variability in olfactory function, body weight, and metabolic factors. Further research is necessary to gain a deeper understanding of the underlying mechanisms and factors contributing to these interconnections. Nevertheless, the targeted inhibition of OB Kv1.3 function represents a promising avenue to treat obesity and metabolic disorders, such as diabetes.

Kv1.3 was initially identified in human T cells in 1984, and its blockers have been investigated as immunomodulators since the mid-1990s. Clofazimine, a representative Kv1.3 inhibitor, has been extensively used in the clinical management of inflammatory dermatological conditions, such as leprosy, and may serve as a model for drug optimization [36,128]. Building on this foundation, drug development efforts have proposed targeting the Kv1.3 channel within the OB as a potential strategy. Gene-targeted deletion of Kv1.3 in mice improved resistance to diet-induced obesity [59]. In another study on diet-induced obese mice, the administration of the Kv1.3 inhibitor margatoxin through OB-targeted osmotic mini-pumps led to a reduction in body weight and an improvement in glucose clearance at a more rapid rate [129]. While olfactory function was not discussed, this finding suggests that the targeted inhibition of Kv1.3 in the OB may offer a promising avenue to treat obesity and related metabolic disorders. Moreover, the channels in M/TCs that exhibit analogous or antithetical effects to those of Kv1.3, such as K-ATP, K2P, and ASICs, warrant further investigation in the context of obesity and metabolism. A deeper understanding of the functions and interactions of these ion channels within the OB may offer valuable insights into the development of more efficacious therapeutic strategies to address these complex and prevalent health conditions.

### 4.2. Nav1.7

Recent studies have elucidated the role of the Na^+^ channel Nav1.7 in both pain perception and olfaction. The wild-type haplotype of SCN9A, which encodes Nav1.7, comprises the rs41268673C>A and rs6746030C>T alleles, and has been associated with reduced pain perception and enhanced olfactory acuity [114,115]. Individuals lacking these alleles exhibited lower pain thresholds and heightened olfactory sensitivity compared to those with the two-allele genotype.

Abnormalities in pain perception and olfaction were more pronounced in patients with genetic mutations. Loss-of-function mutations in SCN9A have been associated with congenital insensitivity to pain and anosmia [102,130], underscoring the potential of Nav1.7 as a genetic marker for these clinical conditions. For example, in mice with a conditional knockout of Nav1.7 OSNs, these neurons generate odor-evoked action potentials but cannot initiate synaptic signaling at the first synapse of the olfactory system [102]. In contrast, a 50-year-old woman with a heterozygous SCN9A mutation (c.3734A>G, p.N1245S) reported burning pain in her feet and abdomen along with hypersensitivity to odors. However, the patient did not respond effectively to treatment with Na^+^ channel inhibitors [116]. Nevertheless, repeated exposure to pleasant odors, including oranges, peaches, and coffee, has been shown to stabilize mood and provide short-term pain relief. These findings highlight the critical role of Nav1.7 in the perception of pain and smell and offer valuable insights for diagnosing related disorders.

The enhanced olfactory sensitivity observed in this case may have contributed to the therapeutic response to odor exposure, potentially explaining its effects on mood and pain relief [116]. This indicates that augmenting the Nav1.7 functionality within the OB could enhance the efficacy of odor therapy for cognitive and mood disorders. Conversely, the inhibition of Nav1.7 in the olfactory system could result in a transient impairment of olfaction, offering a potential method to mask unpleasant odors in specific situations. This could be beneficial for individuals experiencing hyperemesis gravidarum during pregnancy, given the heightened olfactory sensitivity that is a hallmark of this condition [131,132,133]. This approach may have applications for sewage inspectors or cleaners who work in environments with unpleasant odors. The identification of an Na^+^ channel subunit as the causative gene for an inherited form of general anosmia provides new insights into the molecular mechanisms of olfaction.

### 4.3. CNG Channels

As previously described, CNG channel subunits, particularly CNGA2 and CNGA3, play a significant role in odor information processing. Mutations in CNG channel subunits have been linked to several olfactory disorders. An X-linked stop mutation in CNGA2 (c.634C>T, p.R212*) was identified in two brothers and associated with isolated congenital anosmia (ICA), a condition characterized by the inability to smell in otherwise healthy individuals [117]. Magnetic resonance imaging of the brain revealed a reduction in the size of the OBs and flattening of the olfactory sulci, which underscores the essential role of CNGA2 in human olfaction. Subsequently, another stop-gain mutation in CNGA2 (c.577C>T, p.A193*) was identified, resulting in ICA in five affected family members within an Iranian family [118]. Similarly, Cnga2 knockout mice are congenitally anosmic and exhibit markedly impaired olfactory function [134]. These findings indicate that CNGA2 plays a pivotal role in olfactory bulb development and signal transmission. However, given the rarity of CNGA2-related olfactory disorders, with a prevalence of only 1 in 10,000 individuals, the significance of CNGA2 for gene therapy in ICA may be somewhat limited, primarily pertaining to differential diagnosis.

The CNGB1 subunit, which is a component of CNG channels, has been implicated in both photoreceptors and olfactory signal transduction [104,109,119]. The function of CNGB1 is contingent upon its co-expression with a principal subunit (A1–A4), which determines the physical properties of the heteromeric channel, such as ligand sensitivity. This is important for retinal rod phototransduction and odor-induced signal transduction [135]. In a study by Issa et al., nine patients with CNGB1-mutation-associated retinitis pigmentosa were identified, eight of whom exhibited diminished or absent olfactory function [119]. Seven mutations in CNGB1 were identified in patients presenting with hyposmia (*n* = 5) or anosmia (*n* = 3). These observations are consistent with the findings from Cngb1 knockout mice, which demonstrated a reduction in olfactory function [136]. This indicates that CNGB1 mutations may represent a genetic basis for the comorbidity of retinitis pigmentosa and olfactory dysfunction, providing a valuable foundation for the diagnosis of similar disorders.

Similarly, as observed with mutations in Nav1.7 [25] that result in pain and olfactory anomalies, CNGB1 mutations lead to the development of comorbid visual and olfactory impairments. This suggests that ion channels may perform analogous functions across diverse sensory systems and neurons, indicating that when a specific sensory system is influenced by genetic factors, such as ion channel mutations, it may concurrently result in abnormalities in other sensory modalities. These findings provide valuable insights into clinical diagnosis and treatment strategies that can be used.

The development of olfactory diseases is significantly influenced by environmental factors and aging [3,4]. Clinical studies suggest that ion channel disorders, such as ICA, often manifest during childhood due to mutations in CNG channels [118,119]. And concerning the Kv1.3 alleles, many individuals may only become aware of olfactory differences after undergoing olfactory testing, and the influences of age and lifestyle are not well-documented [113,126]. To better elucidate the influence of ion channels in the OB under environmental conditions and aging, further research, including cohort and case–control studies, are warranted.

## 5. Conclusions

In the preceding section, we summarized the findings in the literature regarding the most extensively studied ion channels in M/TCs, GCs, and PGCs within the OB. The current understanding of ion channels in the olfactory system is largely limited by their expression. However, there is a significant gap in our knowledge of their functions. Currently, the primary methods used to investigate the locations and functions of individual ion channels in OB neurons are RNAscope, immunohistochemistry, and electrophysiological recordings. Techniques including single-cell RNA sequencing and spatial transcriptomic analysis may provide further insights into the functional coupling of multiple ion channels in the regulation of OB function. The interactions among ion channels and other signaling molecules during odor stimulation are of significant importance to olfactory research. For instance, further investigation of the interplay between NMDAR and channels such as LTCCs, BK, and Nav in dendrites may provide a more comprehensive understanding of olfactory processing. Given the potential for ion channels to exhibit similar or diverse molecular functions across different cellular structures or neural circuits, it is necessary to use conditional gene editing in conjunction with manipulation by optogenetics or chemogenetics to elucidate the role of individual ion channels within a complex neural circuit.

A significant challenge remains in understanding the contribution of this diversity of ion channels to olfactory processing. The following questions require further investigation. What roles do specific combinations of ion channel subunits and their localization play in shaping the unique responses of OB cells? How do changes in channel function, affected by genetics and drugs, impact olfactory processing? What are the effects of variations in odor exposure, metabolic state, or disease conditions on ion channel expression? Developments of innovative computational and physiological approaches may help to reveal the answers to these questions. Subsequent investigations at the cellular and circuit levels may offer significant insights into the mechanisms of signal transmission within the OB, advancing our understanding of olfactory processes.

## Figures and Tables

**Table 1:** Differential localization and expression of ion channels in the olfactory bulb.

Region	mRNA	Protein	Reference
Glomerular layer			
OSN axons		Kv3.4 (+++), Cav2.1 (+++), Cav2.2 (+++), Nav1.7 (+++)	[37,38,39,40,41]
Periglomerular cells	Kv1.2 (+), Kv1.3 (++), Kv1.4 (++), TASK-1 (+++), TASK-3 (+++), Kv10.1 (±), Kv10.2 (±)	Kv1.3 (++), Kv1.4(++), Kv3.1, Kv4.3 (+++), Kv10.1 (++), Kir2.1 (+++), Kir2.2 (+++), Cav2.1 (++), Cav3.1 (+++), CaV3.3 (+++), CNGA3 (++), KNa1.1 (+++), KNa1.2 (+++)	[33,37,42,43,44,45,46,47,48,49]
Plexiform layer			
TC somata	Kv1.1 (+), Kv1.2 (+++), Kv1.3 (+++), Kv1.4(+++)	Kv1.2 (++), Kv1.4 (+++), Kv10.1 (++), Kir2.4 (++), KNa1.1 (++), CNGA3 (+++)	[37,42,43,44,48,50]
TC dendrites		Kv1.1 (+), Kv1.3 (++), Kv1.4 (+++), Kv4.2 (+++), CNGA3 (+++),	
TC axons		Kv1.2 (+++)	
Mitral cell layer			
MC somata	Kv1.1 (+), Kv1.2 (+++), Kv1.3 (+++), Kv1.4 (+++), Kv10.1 (++), Kv10.2 (+++), KNa1.1 (+++), KNa1.2 (+)	Kv1.2 (+++), Kv1.4 (+++), Kv10.1 (+++), Kir2.1 (+), Kir2.2 (+), Kir2.3 (+), Kir2.4 (++), KNa1.1 (++), KNa1.2 (++), Cav3.1 (+), Cav3.3 (+), CNGA3 (+++)	[32,37,42,43,44,48,50,51,52,53,54]
MC dendrites		Kv1.1 (+), Kv1.3 (++), Kv1.4 (+++), Kv4.2 (+++), Kir2.3 (++), Cav1.2 (+++), Cav2.2, Cav3.1 (++), Cav3.3 (+), CNGA3 (+++), KNa1.1 (++), KNa1.2 (++), KCa2.x	
MC axons		Kv1.2 (+++)	
Granule cell layer			
GC somata	Kv1.1 (++), Kv1.3 (+++), Kv1.4 (+++), Kv1.6 (++), TASK-1 (++), TASK-3 (++), TRAAK (+), TREK-1 (+), TREK-2 (+), TWIK-1 (+), Kv10.1 (++), Kv10.2 (+++), KNa1.1 (+++), KNa1.2 (+)	Kv1.6 (few), Kv4.2 (++), Kv4.3 (++), Kv10.1 (++), Kir2.1 (++), Kir2.3 (+), Kir2.4 (few), Cav3.1 (++), CaV3.3 (++), Nav1.2 (+++)	[32,37,40,42,44,45,49,52,55,56,57,58]
GC dendrites		Kv1.1 (++), Kv1.3 (+++), Kv1.4 (+++), Kv4.2 (+++), KCa1.1, KNa1.1 (++), KNa1.2 (++), Cav3.1 (++). Cav3.3 (++), Nav1.2 (+++)	

Relative expression intensities are graded: +++, intense; ++, moderate; +, weak; ±, just above background; few, few cells are strongly positive.

## Data Availability

Data sharing is not applicable.

## Abbreviations

OB	Olfactory bulb
TRP	Transient receptor potential
BK	Large conductance K^+^ channels
Kv	Voltage-gated K^+^ channels
CNG	Cyclic nucleotide-gated
Cav	Voltage-gated Ca^2+^ channels
OSN	Olfactory sensory neuron
GL	Glomerular layer
EPL	External plexiform layer
MCL	Mitral cell layer
IPL	Internal plexiform layer
GCL	Granule cell layer
M/TC	Mitral/tufted cell
GABA	γ-aminobutyric acid
GC	Granule cell
PGC	Periglomerular cell
Ca^2+^/CaM	Ca^2+^/calmodulin
ICA	Isolated congenital anosmia
Kir	Inward rectifier K^+^ channels
ATP	Adenosine triphosphate
K-ATP	ATP-sensitive K^+^ channels
K2P	Two-pore domain K^+^ channels
DRG	Dorsal root ganglion
A1R	Adenosine A1 receptors
KCa	Ca^2+^-activated K^+^ channels
SK	Small conductance K^+^ channels
NMDAR	N-methyl-D-aspartate receptors
KNa	Na+-activated K^+^ channels
P/QTCCs	P/Q-type Ca^2+^ channels
NTCCs	N-type Ca^2+^ channels
LTCCs	L-type Ca^2+^ channels
TTCCs	T-type Ca^2+^ channels
RTCCs	R-type Ca^2+^ channels
Nav	Voltage-gated Na^+^ channels
cAMP	Cyclic adenosine monophosphate
cGMP	Cyclic guanosine monophosphate
ASICs	Acid-sensing ion channels
